# Supplementary material for: Optimizing the colorectal cancer screening programme using faecal immunochemical test (FIT) in Flanders, Belgium from the “interval cancer” perspective
Source: Br J Cancer. 2022 Jan 12;126(7):1091–9. doi: 10.1038/s41416-021-01694-2 (PMC8980044; doi:10.1038/s41416-021-01694-2)
Supplement: Supplementary file 1 — Supplementary material [file 41416_2021_1694_MOESM1_ESM.docx]

# Supplemental material for

## Optimizing the colorectal cancer screening programme using faecal immunochemical test (FIT) in Flanders, Belgium from the “interval cancer” perspective

## Supplementary tables

**Supplementary Table 1** The STROBE checklist for observational studies in epidemiology applied in this study.

**Supplementary Table 2** Distributions of quantitative results of faecal immunochemical test (FIT) among FIT-interval cancers, stratified by the first/second year of the screening interval, genders, age groups, tumour stages and tumour locations.

### **Supplementary Table 1** The STROBE research checklist for observational studies in epidemiology applied in this study.

|  | Item No | Recommendation |
| --- | --- | --- |
| **Title and abstract** | 1 | Title  *Optimizing the colorectal cancer screening programme using faecal immunochemical test (FIT) in Flanders, Belgium from the “interval cancer” perspective* |
|  |  | Provide in the abstract an informative and balanced summary of what was done and what was found  ***Background***  *Interval cancer (IC) is a critical issue in colorectal cancer (CRC) screening. We identified factors associated with ICs after faecal immunochemical test (FIT) screening and explored the impact of lowering FIT cut-off or shortening screening interval on FIT-ICs in Flanders.*  ***Methods***  *FIT participants diagnosed with a CRC during 2013 2018 were included. Factors associated with FIT ICs were identified using logistic regression. Distributions of FIT results among FIT-ICs were examined.*  ***Results***  *In total, 10,122 screen-detected CRCs and 1,534 FIT-ICs were included (FIT-IC proportion of 13%). FIT-ICs occurred more frequently in women (OR 1.58 [95% CI 1.41–1.76]) and ages 70–74 (OR 1.35 [1.14–1.59]). FIT-ICs were more often right sided (OR 3.53 [2.98–4.20]), advanced stage (stage IV: OR 7.15 [5.76–8.88]), and high grade (poorly/undifferentiated: OR 2.57 [2.08–3.18]). The majority (83–92%) of FIT ICs would still be missed if FIT cut-off was lowered from 15 to 10 µg Hb/g or screening interval was shortened from two to one year.*  ***Conclusions***  *FIT-ICs were more common in women, older age, right sided location, advanced stage and high grade. In Flanders, lowering FIT cut off (to 10 µg Hb/g) or shortening screening interval (to one year) would have a minimal impact on FIT-ICs.* |
| Introduction | | |
| Background/rationale | 2 | Explain the scientific background and rationale for the investigation being reported  *Worldwide, colorectal cancer (CRC) accounts for one in every ten cancer cases and deaths. Between 2012 and 2018, the number of patients diagnosed with CRC in Europe increased from 447,000 to 500,000 while the number of those who died from this disease increased from 215,000 to 242,000.^1^ In Flanders (57% of the Belgian population), CRC is the second most common cancer in women and third in men. In 2018, the age-standardized (world standard population) CRC incidence rates for men and women were 33.8/100,000 person-years and 24.1/100,000 person-years, respectively.^2^*  *CRC screening helps to detect precancerous lesions and tumours at an early stage and can therefore reduce CRC-related mortality. Faecal occult blood test is recommended for organised CRC screening by the European guidelines.^3^ Guaiac faecal occult blood test (gFOBT) has been shown to reduce CRC-related mortality by 15.0–33.0%.^4-6^ In recent years, faecal immunochemical test (FIT) is a more preferred screening test by many CRC screening programmes since it offers a higher sensitivity compared to gFOBT.^7^ Among the organised screening programmes that use FIT, each programme implements a different screening strategy: a different FIT cut-off (15–80 µg Hb/g) or screening interval (one-year or two-year), depending on its desired diagnostic values and capacity of follow-up colonoscopy after a positive FIT.^7^ Research is still ongoing to identify the optimal screening strategy for each programme.*  *The optimization of a screening programme needs to be approached from different angles. The occurrence of FIT interval cancers (FIT-ICs) is an important quality indicator of any screening programme using FIT. FIT-IC is defined as CRC diagnosed after a negative FIT and before the next recommended examination.^3^ The proportion of FIT-ICs ranged from 7% to 51% in previous studies with a FIT cut-off between 10 and 80 µg Hb/g (two-year screening interval).^8-13^ In addition, FIT-ICs have been shown to be associated with more advanced stage, higher grade and more aggressive histotype, resulting in reduced survival compared to screen-detected CRCs.^8,12,14,15^ The European guidelines recommend monitoring interval cancers as a parameter of programme effectiveness.^3^*  *Prior research has pointed out several subgroups who are at a higher risk of having FIT-IC such as women^9,13,16-19^ and older age.^16,20^ These individuals may be disadvantaged when only a single FIT cut-off is used for the whole screening population. Therefore, many studies have advocated individualizing FIT usage in CRC screening to increase equity across subgroups and improve the performance of the screening programmes.^13,21-23^ Gender-specific FIT cut-offs have been introduced in several screening programmes to narrow the gap between men and women in the test’s diagnostic performance, especially sensitivity, such as 80 µg Hb/g for men and 40 µg Hb/g for women in Sweden,^24,25^ or 70 µg Hb/g for men and 25 µg Hb/g for women in Finland^26^. Shortening screening interval has also been suggested as a possible measure for subgroups with a lower FIT sensitivity.^21^*  *In Flanders, the organised CRC screening programme has been in place since 2013, which offers a free biennial FIT to all individuals in the target population using a centralized invitation procedure. During the study period (2013–2018), the programme used a uniform FIT cut-off of 75 ng Hb/ml (15 µg Hb/g) and a uniform screening interval (two‑year) for all screening individuals aged 53-74 years. However, like other CRC screening programmes, the Flemish programme is attempting to determine the optimal screening strategy to optimize its efficacy.* |
| Objectives | 3 | State specific objectives, including any prespecified hypotheses  *The objectives of the current study were to identify factors associated with the risk of having a FIT-IC versus a screen-detected CRC and explore the impact of lowering FIT cut-off or shortening screening interval on reducing FIT-ICs in the context of the Flemish CRC screening programme.* |
| Methods | | |
| Study design | 4 | Present key elements of study design early in the paper  *An observational study using data on screen-detected CRCs and FIT‑ICs between October 2013 (start of the programme) and December 2018 (the latest year for which all required data were complete).* |
| Setting | 5 | Describe the setting, locations, and relevant dates, including periods of recruitment, exposure, follow-up, and data collection  ***Flanders and its CRC screening programme***  *Flanders is the most populated region of Belgium (6.6 million inhabitants, 57% of Belgian population).^27^ The CRC screening programme in Flanders has been in place since October 2013 and offers a free biennial quantitative FIT (OC-sensor, Eiken Chemical Co, Tokyo, Japan) to all citizens eligible for CRC screening. During the study period, the target screening ages were extended gradually from 56–74 in 2013 to 53–74 in 2018 (up to 50–74 in 2020).* *People were excluded from the screening invitation list if they had had a stool test in the past two years, a virtual colonoscopy in the past four years or a complete colonoscopy in the past ten years, had been diagnosed with CRC in the past ten years or had had a colectomy (excluded permanently). The positivity cut-off of FIT was ≥15 µg Hb/g [or 75 ng Hb/ml, conversion formula: µg Hb/g faeces = (ng Hb/ml buffer) × 2 mL buffer / 10 mg faeces collected].* *In 2018, the response rate of the Flemish CRC screening programme was 51.5% (~670,000 invitations sent out). The FIT sensitivity values were 72.4% and 86.3%, positive predictive values were 3.7% and 4.1%, and detection rates were 0.17% and 0.19%, respectively, for invasive and in situ cancers.^28^*  *After a positive FIT, patients are advised to undergo a colonoscopy ordered either through their GP or by consulting a gastroenterologist. During the study period, follow-up colonoscopy was not included as part of the population-based CRC screening programme in Flanders and* *there was also no centralised colonoscopy quality register in Belgium. Data on the performance of a follow-up colonoscopy after a positive FIT, based on the reimbursement data from health insurance companies, was available at the Belgian Cancer Registry and used to create an exclusion list (as detailed above) for the CRC screening programme for the next invitation round.^16^*  ***Study population and data sources***  *The study population included all eligible individuals for CRC screening (53–74 years) who participated in the Flemish CRC screening programme between October 2013 (start of the programme) and December 2018 (the latest year for which all required data were complete) and were subsequently diagnosed with either a screen-detected CRC or FIT-IC in the same period.* |
| Participants | 6 | Give the eligibility criteria, and the sources and methods of selection of participants. Describe methods of follow-up  *A screen-detected CRC and FIT-IC were defined by the screening programme as follows:*  *- Screen-detected CRC was defined as a CRC diagnosed after a positive FIT, within six months after the first follow-up colonoscopy and before the next recommended FIT invitation (24 months).*  *- FIT-IC was defined as a CRC diagnosed after a negative FIT and before the next recommended FIT invitation (24 months).*  *Data on individuals’ screening history (FIT result and follow-up colonoscopy) were retrieved from the database of the Flemish Centre for Cancer Detection and were linked with data on tumour characteristics (location, stage and differentiation grade) from the population-based Belgian Cancer Registry. The Belgian Cancer Registry collects information regarding new CRC diagnoses based on obligatory notifications provided by the oncological care programmes and the laboratories for pathological anatomy. Validated data are currently available for Flanders from 2001–2018 with an estimated >98% completeness. In the case of multiple lesions, only the most advanced finding was retained (e.g., prioritizing invasive lesions over in situ lesions). The applicable TNM edition at the time of diagnosis was used (TNM 7^th^ edition for incidence years 2013–2016 and TNM 8^th^ edition for incidence years 2017–2018).^29,30^ A combined TNM stage was determined by prioritizing pathological staging over clinical staging, except in the presence of clinical distant metastases which were always considered stage IV. Tumour location was classified as right side (from the cecum to the transverse colon), left side (from the splenic flexure to the sigmoid colon) or rectum.^8,9,18^ Differentiation grade was classified as well‑differentiated (grade 1), moderately‑differentiated (grade 2) and poorly/undifferentiated (grade 3–4).^8,14,31,32^* |
|  |  | (*b*) For matched studies, give matching criteria and number of exposed and unexposed: *Not applicable* |
| Variables | 7 | Clearly define all outcomes, exposures, predictors, potential confounders, and effect modifiers.  *Exposures:*  *Individuals’ characteristics*  *Gender*  *Age at FIT screening*  *Tumours’ characteristics*  *Location*  *Stage*  *Differentiation grade*  *Outcomes: The risk of having a FIT-IC versus a screen-detected CRC*  *Covariates*  *For the variables “gender” and “age at FIT screening” that were assessed at the time of FIT screening, “age at FIT screening” and “gender” were adjusted for in the multivariable analyses, respectively.*  *For the variables “location”, “stage” and “differentiation grade” that were assessed at the time of cancer diagnosis, age at cancer diagnosis and gender were adjusted for in the multivariable analyses.* |
| Data sources/ measurement | 8* | For each variable of interest, give sources of data and details of methods of assessment (measurement). Describe comparability of assessment methods if there is more than one group  ***Sources of data***  *Data on individuals’ screening history (FIT result and follow-up colonoscopy) were retrieved from the database of the Flemish Centre for Cancer Detection and were linked with data on tumour characteristics (location, stage and differentiation grade) from the population-based Belgian Cancer Registry. The Belgian Cancer Registry collects information regarding new CRC diagnoses based on obligatory notifications provided by the oncological care programmes and the laboratories for pathological anatomy. Validated data are currently available for Flanders from 2001–2018 with an estimated >98% completeness. In the case of multiple lesions, only the most advanced finding was retained (e.g., prioritizing invasive lesions over in situ lesions).*  ***Exposures***  *The applicable TNM edition at the time of diagnosis was used (TNM 7^th^ edition for incidence years 2013–2016 and TNM 8^th^ edition for incidence years 2017–2018).^29,30^ A combined TNM stage was determined by prioritizing pathological staging over clinical staging, except in the presence of clinical distant metastases which were always considered stage IV. Tumour location was classified as right side (from the cecum to the transverse colon), left side (from the splenic flexure to the sigmoid colon) or rectum.^8,9,18^ Differentiation grade was classified as well‑differentiated (grade 1), moderately‑differentiated (grade 2) and poorly/undifferentiated (grade 3–4).^8,14,31,32^*  ***Outcomes***  *A screen-detected CRC and FIT-IC were defined by the screening programme as follows:*  *- Screen-detected CRC was defined as a CRC diagnosed after a positive FIT, within six months after the first follow-up colonoscopy and before the next recommended FIT invitation (24 months).*  *- FIT-IC was defined as a CRC diagnosed after a negative FIT and before the next recommended FIT invitation (24 months).* |
| Bias | 9 | Describe any efforts to address potential sources of bias  *Administrative data were used for all the study variables, thus eliminating the common selection and recall bias associated with self-reported data.* |
| Study size | 10 | Explain how the study size was arrived at  *All 11,656 FIT participants between October 2013 and December 2018 who were subsequently diagnosed with either a screen-detected CRC (N=10,122) or FIT-IC (N=1,534) in the same period were included for all the analyses; except in the analyses regarding tumour location where we only included 10,111 screen-detected CRCs and 1,528 FIT-ICs because two CRCs with an overlapping location and 15 CRCs in the right side of the colon but detected with an incomplete colonoscopy were removed.* |
| Quantitative variables | 11 | Explain how quantitative variables were handled in the analyses.  *Age at FIT screening was categorized into three groups: 53-59, 60-69 and 70-74. Other variables were originally categorical.* |
| Statistical methods | 12 | Describe all statistical methods, including those used to control for confounding  *Continuous variables were described with medians (interquartile ranges) and categorical variables were described with numbers (percentages). Logistic regression was used to assess the associations between individuals’ and tumours’ characteristics and the risk of having a FIT-IC versus a screen-detected CRC. Crude and adjusted odds ratios (for age and gender) with 95% confidence intervals were reported. In this study, stage I was used as the reference to enable the comparison with other studies where only stages I–IV were included.^8,10-12,31^ FIT-IC proportions for different profiles combining individuals’ and tumours’ characteristics were presented. FIT-IC proportion was calculated as the number of FIT-ICs divided by the total number of FIT-ICs and screen-detected CRCs and presented as percentage to enhance comprehension.^11,12,16^ We also examined the distributions of FIT results among FIT-ICs in the first/second year of the screening interval and by patients’ and tumours’ characteristics to explore the impact of shortening FIT screening interval or lowering FIT cut-off on reducing FIT-ICs. There is discrepancy among guidelines regarding whether to include in situ cancers in the definition of colorectal carcinoma (TNM and Japanese classification systems) or not (European and US classification systems).^3,30,33^ To facilitate the comparison of our findings with those of other studies, we present, where it is possible, the results for in situ cancers as a separate group from invasive cancers.*  *P-values less than 0.05 (two‑sided) were considered statistically significant. All analyses were performed with RStudio (version 1.3.1056; RStudio, PBC, Boston, MA).* |
|  |  | Describe any methods used to examine subgroups and interactions: *Not applicable.* |
|  |  | Explain how missing data were addressed:  *Data on gender, age at FIT screening and cancer diagnosis were known for all study subjects. About 14% of the tumours had an unknown stage, 38% had an unknown location and 47% had an unknown differentiation grade. In such cases, the data providers (oncological care programmes and/or laboratories for pathological anatomy) filled in the variables with an unspecified code or left them blank (although the fields were mandatory in the registration form). In our data analyses, these observations were included under the “unknown” category.* |
|  |  | If applicable, explain how loss to follow-up was addressed: *Not applicable.* |
|  |  | Describe any sensitivity analyses: *Not applicable.* |
| Results | | |
| Participants | 13* | Report numbers of individuals at each stage of study:  *In total, 11,656 CRCs diagnosed after FIT screening were included, with a FIT-IC proportion of 13%. The number of CRCs decreased gradually each year from 3,174 in 2014 to 1,524 in 2018.* |
|  |  | Give reasons for non-participation at each stage: *Not applicable.* |
|  |  | Consider use of a flow diagram: *Not applicable.* |
| Descriptive data | 14* | Characteristics of study participants:  *Most of the study subjects were male (64.5%). The median age at FIT screening was 66 years. A large proportion of the tumours were classified as “unknown” for location (38.3%), stage (14.0%) or differentiation grade (46.7%). Among the tumours with known categories, the majority presented in the left side of the colon or rectum (5,680/7,188; 79.0%), at stage I or in situ (7,184/10,019; 72.0%) and were moderately differentiated (3,577/6,209; 57.0%) (****Table 1****).* |
|  |  | Number of participants with missing data:  *Data on gender, age at FIT screening and cancer diagnosis were known for all study subjects. About 14% of the tumours had an unknown stage, 38% had an unknown location and 47% had an unknown differentiation grade. In such cases, the data providers (oncological care programmes and/or laboratories for pathological anatomy) filled in the variables with an unspecified code or left them blank (although the fields were mandatory in the registration form). In our data analyses, these observations were included under the “unknown” category.* |
|  |  | Follow-up time: *between October 2013 and December 2018* |
| Outcome data | 15* | Report numbers of outcome events or summary measures over time:  *In total, 11,656 CRCs diagnosed after FIT screening were included, with a FIT-IC proportion of 13% (Table 1).* |
| Main results | 16 | *(*a) Give unadjusted estimates and, if applicable, confounder-adjusted estimates and their precision (eg, 95% confidence interval).  ***Factors associated with the risk of having a FIT-IC versus a screen-detected CRC***  *The risk of having a FIT-IC versus a screen-detected CRC was 1.6 times higher in women vs. men (OR = 1.58 [1.41–1.76]) and 1.4 times higher in people aged 70–74 compared to ages 53-59 (OR = 1.35 [1.14–1.59]). Regarding tumours’ characteristics, the risk of having FIT-IC was 3.5 times higher for tumours in the right side of the colon (OR = 3.53 [2.98–4.20]) and twice higher for those in the rectum (OR = 2.01 [1.72–2.37]) compared to those in the left side; 7.2 times higher for stage IV compared to stage I (OR = 7.15 [5.76–8.88]); and 2.6 times higher for poorly/undifferentiated lesions compared to well-differentiated lesions (OR = 2.57 [2.08–3.18]) (****Table 2****).*  ***Figure 1*** *illustrates the increase in FIT-IC proportion when tumour stage (the strongest factor associated with an increased risk of having a FIT-IC) was combined with other factors. FIT-IC proportion was 45% when considering stage IV alone but increased to 49% when stage IV was combined with age 70–74; 55–56% when stage IV was combined with female gender or poorly/undifferentiated grade and to 63% when stage IV was combined with right side location.*  ***Distribution of FIT results among FIT-ICs in the first/second year of screening interval and by patients’ and tumours’ characteristics***  *The number of FIT-ICs increased during the two-year screening interval (****Figure 2****). More than sixty percent (922/1,534) of FIT-ICs were detected in the second year. According to the distributions of FIT results (****Figure 3*** *and* ***Supplementary Table 1****), 83-92% FIT-ICs in both sexes, all age groups, tumour locations and stages had a low quantitative FIT result of ≤ 10 µg Hb/g. This majority of FIT‑ICs would not be picked up if the FIT cut-off was lowered from 15 to 10 µg Hb/g. Similarly, 89% of the FIT-ICs detected in the second year of the two-year screening interval also had a low FIT result of ≤ 10 µg Hb/g, suggesting that most FIT-ICs would still be missed even if the screening interval was shortened from two to one year, given the current FIT cut-off of 15 µg Hb/g.*  Make clear which confounders were adjusted for and why they were included  *For the variables “gender” and “age at FIT screening” that were assessed at the time of FIT screening, “age at FIT screening” and “gender” were adjusted for in the multivariable analyses, respectively.*  *For the variables “location”, “stage” and “differentiation grade” that were assessed at the time of cancer diagnosis, age at cancer diagnosis and gender were adjusted for in the multivariable analyses.* |
|  |  | Report category boundaries when continuous variables were categorized: *Not applicable.* |
|  |  | If relevant, consider translating estimates of relative risk into absolute risk for a meaningful time period: *Not applicable.* |
| Other analyses | 17 | Report other analyses done—eg analyses of subgroups and interactions, and sensitivity analyses: *Not applicable.* |
| Discussion | | |
| Key results | 18 | Summarise key results with reference to study objectives  *Using data of all screen-detected CRCs and FIT-ICs diagnosed in FIT participants for CRC screening in Flanders during 2013–2018, we identified several factors associated with a higher risk of having a FIT-IC versus a screen-detected CRC. These include female gender, older age, right side and rectum locations, advanced stage and high grade. The majority (83–92%) of FIT-ICs in both the first and second year of the two-year screening interval and all subgroups had a low FIT result of ≤ 10 µg Hb/g, indicating a minimal impact of shortening the screening interval from two years to one year or lowering the FIT cut-off from 15 µg Hb/g (the current cut‑off) to 10 µg Hb/g on reducing FIT-ICs.* |
| Limitations | 19 | Discuss limitations of the study, taking into account sources of potential bias or imprecision. Discuss both direction and magnitude of any potential bias  *Several limitations need to be acknowledged. Firstly, since data on both FIT participation and cancers were retrieved between October 2013 and December 2018, data on screen-detected CRCs and FIT‑ICs following FITs taken in the latest years (2017–2018) might be incomplete, resulting in an underestimation of both screen‑detected CRC and FIT‑ICs, with an expected larger extent for FIT‑ICs. As a result, FIT‑IC proportion might be underestimated, especially for the latest screening years. Secondly, the administrative data used in this study contained sizable proportions of tumours with unknown location, stage or differentiation grade. Future research can benefit from using pathology reports to supplement missing or non-specific information. Lastly, data on molecular characteristics of tumours are lacking in the current study. We plan to analyse pathology reports to study the difference in molecular characteristics between screen-detected CRCs and FIT‑ICs, especially those at an advanced stage, in the next step.* |
| Interpretation | 20 | Give a cautious overall interpretation of results considering objectives, limitations, multiplicity of analyses, results from similar studies, and other relevant evidence:  *The FIT-IC proportion in Flanders during 2013–2018 was 13%, which lies within the range of FIT-IC proportion of 7–23% in other screening programmes using a FIT cut-off between 10 and 20 µg Hb/g.^8-12^ Our study also supports previous findings that ICs after a negative faecal occult blood test are more common in women, ^9,13,16-19^ older people,^16,20,36^ in the right side of the colon^8,9,11,16-19,22^ or in the rectum,^10,17,37^ at a more advanced stage^8-14,16,22,31,37^ and with a higher grade,^14,31^ compared to screen-detected CRCs. Prior literature has proposed several explanations for these associations but definitive conclusions have not been reached.*  *The fact that women have a higher risk of having FIT-IC than men might be due to lower blood haemoglobin concentrations,^17,20,38^ a longer colonic transit time leading to a greater degree of haemoglobin degradation,^39^ or a higher proportion of harder-to-detect, right-sided cancers.^17,20,22,40^ The last proposed reason might contribute modestly to the explanation since after adjusting for tumour location, we found almost the same association between gender and the risk of having a FIT-IC versus a screen-detected CRC (OR 1.53 [1.36–1.71]), showing 1.53 times higher risk of having a FIT‑IC in women, independently of location.*  *Although a number of studies have shown a lower FIT sensitivity in older people,^16,20,36^ no possible explanations for this association have been given. Fraser et al (2014) reported increasing faecal haemoglobin concentrations with age (50–69 years) for both men and women in the screening populations for CRC.^39^ With a higher faecal haemoglobin concentration and a higher CRC incidence rate, we would normally expect a higher FIT sensitivity in the older age group; it is interesting that studies have found the inverse. We also tested the possibility that CRCs diagnosed in older people presented at a more advanced stage and therefore were missed more by FIT. However, this hypothesis was not supported by our data since the association between the oldest age group (70–74) and the risk of having a FIT-IC versus a screen-detected CRC remained (OR 1.31 [1.11–1.56]) after we adjusted for tumour stage in addition to gender. It is a question of future research to investigate the possible reasons for the lower sensitivity of FIT in the older age group.*  *Prior research has reported a higher proportion of nonpolypoid (flat) tumours in the right side compared to the left side of the colon.^21,41^ These tumours tend to have a higher risk of malignant transformation and invasiveness at a relatively smaller size.^41,42^ Due to the smaller areas in contact with faeces and sparser vasculature in the mucosa, they bleed less and are less sensitive to FIT.^21,43^ A longer transit time from the right side may also lead to a greater degree of haemoglobin degradation, and therefore more false‑negative results occur with right‑sided tumours.^17,19,21,44^ Selby et al (2018) reported a significantly lower faecal haemoglobin level of the right‑sided cancers among FIT screenees compared to that of the left‑sided cancers (12.4 versus 60.0 μg Hb/g; p < 0.001).^20^*  *Regarding a higher risk of being a FIT-IC for tumours in the rectum compared to the left side location, erythrocytes in blood released from rectum lesions may not have been sufficiently haemolysed during a short passage through the rectum, and therefore do not yield a positive result to a FIT.^13,37^ In the same study by Selby et al, the faecal haemoglobin level of the rectal cancers was also significantly lower than that of the left-sided cancers (24.4 versus 60.0 μg Hb/g, p < 0.001).^20^ Another possible explanation is that rectal bleeding more often presents with bright red blood in faeces, which is easier to notice. Screening participants generally have a heightened awareness of signs of blood in their faeces. Once they notice bright red blood in faeces, they tend to consult with primary care promptly.^22^ It cannot be ruled out that low FIT effectiveness for tumours in the right colon or rectum may also stem from lesions that grow more rapidly.^41,42,45^*  *Compared to screen-detected CRCs, FIT‑ICs exhibited a higher grade and aggressive histotype (signet ring cell and mucinous carcinomas).^14^ However, it is still unclear to what degree FIT‑ICs are due to FIT false‑negative tests or a faster growth pathway of high-grade and aggressive histotype tumours. A recent study by Steel et al reported a mean time of 10.9 ± 2.9 months from FIT screening to diagnoses of all ICs and 11.6 ± 7.2 months from FIT screening to diagnoses of high‑grade and aggressive histotype ICs.^14^ The more advanced stage of FIT‑ICs compared to screen‑detected CRCs might be because FIT is known to be less sensitive for flat, right-sided and poorly or undifferentiated lesions,^14,46-48^ tumours with these characteristics may be missed more often by FIT. Once these tumours are detected later on when symptoms appear, they are already at an advanced stage. A proportion of these ICs may also originate from tumours with more aggressive characteristics and worse behaviour, which actually arose after a true negative FIT.^14,15^ Our findings reinforce the current advice to the screening participants not to regard a negative FIT result as a “certificate of health”. Instead, they need to be vigilant and seek primary care promptly when any signs or symptoms appear.^22,49,50^*  *To reduce FIT-ICs, previous studies have also suggested personalizing FIT cut‑offs, for example, using a lower cut-off in the subgroups with a lower FIT sensitivity such as women and older people.^13,21-23^ However, our data showed that in Flanders, lowering FIT cut‑off from 15 to 10 μg Hb/g would only have a limited impact on reducing FIT‑ICs since more than 83% of FIT‑ICs had a low FIT result of ≤ 10 μg Hb/g across genders, age groups, tumour locations and stages. Although the FIT test used in the Flemish screening programme could theoretically detect up to 3 μg Hb/g faeces, the quantitative results between 3–10 μg Hb/g were considered (quantitatively) unreliable due to large deviations. Therefore, lowering FIT cut‑off to below 10 μg Hb/g would not be a suitable option for the test used. Prior research has reported around 75% of FIT‑ICs with a low level of haemoglobin (< 10 μg Hb/g) and 19.4–44% with an undetectable level (0 μg Hb/g).^11-13^ This implies that the majority of FIT‑ICs would still be missed even with a drastic reduction in the FIT cut‑off.*  *Gender-specific cut-offs have also been introduced/piloted in several screening programmes, for example, 40 µg Hb/g for women and 80 µg Hb/g for men in Sweden;^24,25^ or 25 µg Hg/g for women and 70 µg Hg/g for men in Finland.^26^ In Flanders, a much lower FIT cut‑off of 15 µg Hg/g has already been applied for all screening individuals. Our results suggest that 15 µg Hg/g should be the lowest FIT cut‑off that a CRC screening programme should aim for, regardless of the patient’s gender and age. This recommendation is supported by a recent study by Vanaclocha-Espi et al (2021) which found the optimal FIT cut‑off of around 15 µg Hg/g for the subgroup of women aged 60–69, which had the lowest FIT sensitivity among the subgroups evaluated (women and men, aged 50–59 and 60–69).^51^*  *Many studies have also highlighted a substantial increase in colonoscopy demand for only a marginal gain in sensitivity by lowering FIT cut‑off.^13,20,52^ For example, the Scottish Bowel Screening Programme reported that halving the FIT cut‑off in their programme from 80 μg Hb/g to 40 μg Hb/g faeces would reduce the FIT‑IC proportion from 50.8% to 45.9%, but would increase the number of colonoscopies required by 58.6%.^13^ An American screening programme predicted that lowering the FIT cut‑off in their programme from 20 μg/g to 10 μg/g would increase the programme’s sensitivity by only 3% (from 76.3% to 79.3%) while increasing the number of positive results per one cancer case detected from 52 to 85.^20^*  *In agreement with Giorgi Rossi et al,^18^ we also observed more FIT‑ICs in the second year than the first year of the two‑year screening interval (60% of all ICs). The difference in the number of FIT-ICs between the second and the first year was 310 cases. It seems, at first glance, that shortening the FIT screening interval from two to one year during the study period might have helped to reduce this number of FIT‑ICs.^21^ However, 89% of FIT‑ICs in the second year were found to have a low FIT result at screening of ≤ 10 µg Hb/g, suggesting that the majority of ICs would still be missed even when the screening interval was shortened to one year. One might also argue that the FIT results were obtained at the time of screening and as tumours progressed between the first and second year, FIT results might get higher and reach the positive cut‑off. The proportion of such tumours seemed to be modest since our data showed that 83–92% of FIT‑ICs across all tumour stages had a low FIT result of ≤ 10 µg Hb/g. Thus, in the programmes where a low FIT cut‑off of 15 µg Hb/g is implemented, shortening screening interval from two to one year seems to produce only a marginal impact on reducing FIT-ICs.*  *Meanwhile, a CRC screening programme should also consider the impact of screening interval on screen-detected CRCs and the related costs. Specifically, when the screening interval of a two-year programme was shortened to one year, the number of prevalent cases detected among individuals entering the screening programme for the first time would be almost similar (same population and same target screening ages). The main difference is that after the first screening round, the population would repeat screening right the next year in the one-year programme, instead of waiting for two years in the two-year programme. A proportion of the screen-detected incident CRCs in the two-year programme would be diagnosed one year earlier in the one-year programme. This, however, comes at the cost of having the whole screening population undergo FIT every year instead of every two years (Flanders: ~850,000 individuals in 2020),^28^ meaning doubling the entire process of screening invitation, FIT provision, result analyses and follow‑up with colonoscopy after a positive FIT.*  *With data from a population‑based screening programme of the largest region in Belgium, which used a FIT cut-off within the common range of 10–20 µg Hb/g, our results can be widely generalized to other CRC screening programmes. The large sample size allowed us to stratify our results by multiple participants’ and tumours’ characteristics.* |
| Generalisability | 21 | Discuss the generalisability (external validity) of the study results  *With data from a population‑based screening programme of the largest region in Belgium, which used a FIT cut-off within the common range of 10–20 µg Hb/g, our results can be widely generalized to other CRC screening programmes.* |
| Other information | | |
| Funding | 22 | Give the source of funding and the role of the funders for the present study and, if applicable, for the original study on which the present article is based  *The authors received no specific funding for this work.* |

*Give information separately for exposed and unexposed groups.

**Note:** An Explanation and Elaboration article discusses each checklist item and gives methodological background and published examples of transparent reporting. The STROBE checklist is best used in conjunction with this article (freely available on the Web sites of PLoS Medicine at http://www.plosmedicine.org/, Annals of Internal Medicine at http://www.annals.org/, and Epidemiology at http://www.epidem.com/). Information on the STROBE Initiative is available at http://www.strobe-statement.org.

**Supplementary Table 2** Distributions of quantitative results of faecal immunochemical test (FIT) among FIT-interval cancers, stratified by the first/second year of the screening interval, genders, age groups, tumour stages and tumour locations.

|  | **FIT-ICs (µg Hb/g)** | | | | **Screen-detected cancers** |
| --- | --- | --- | --- | --- | --- |
|  | **≤10** | **>10 to ≤12** | **> 12 to <15** | **<15 (negative)** | **≥15 (positive)** |
| ***Year*** | | | | | |
| Year 1 | 525 (85.8%) | 33 (5.4%) | 54 (8.8%) | 612 (100%) | 9925 |
| Year 2 | 817 (88.6%) | 39 (4.2%) | 66 (7.2%) | 922 (100%) | 197 |
| ***Gender*** | | | | | |
| Male | 750 (88.7%) | 39 (4.6%) | 57 (6.7%) | 846 (100%) | 6670 |
| Female | 592 (86%) | 33 (4.8%) | 63 (9.2%) | 688 (100%) | 3452 |
| ***Age at FIT screening*** | | | | | |
| 53–59 | 211 (89.8%) | 9 (3.8%) | 15 (6.4%) | 235 (100%) | 1800 |
| 60–69 | 705 (86.6%) | 36 (5.5%) | 66 (7.9%) | 807 (100%) | 5483 |
| 70–74 | 426 (87.6%) | 27 (4.2%) | 39 (8.2%) | 492 (100%) | 2839 |
| ***Tumour stage*** | | | | | |
| In situ | 362 (88.3%) | 20 (4.9%) | 28 (6.8%) | 410 (100%) | 4060 |
| Stage I | 243 (88.0%) | 11 (4.0%) | 22 (8.0%) | 276 (100%) | 2438 |
| Stage II | 156 (87.2%) | 11 (6.1%) | 12 (6.7%) | 179 (100%) | 881 |
| Stage III | 206 (86.6%) | 12 (5.0%) | 20 (8.4%) | 238 (100%) | 1031 |
| Stage IV | 189 (82.9%) | 10 (4.4%) | 29 (12.7%) | 228 (100%) | 278 |
| Unknown stage | 186 (91.6%) | 8 (3.9%) | 9 (4.4%) | 203 (100%) | 1434 |

### FIT, Faecal immunochemical test; IC, Interval cancer
